# Topological Properties of Chemical Bonds from Static and Dynamic Electron Densities

**DOI:** 10.1002/zaac.201200535

**Published:** 2013-07-23

**Authors:** Siriyara Jagannatha Prathapa, Jeanette Held, Sander van Smaalen

**Affiliations:** [a] Laboratory of Crystallography, University of BayreuthUniversitaetsstrasse 30, 95447 Bayreuth, Germany

**Keywords:** Amino acids, Electron densities, Chemical bonding, Proteins, X-ray diffraction

## Abstract

Dynamic and static electron densities (EDs) based on the independent spherical atom model (IAM) and multipole (MP) models of crambin were successfully computed, holding no series-termination effects. The densities are compared to EDs of small biological molecules at diverse temperatures. It is outlined that proteins exhibit an intrinsic flexibility, present as frozen disorder at 100 K, in contrast to small molecules. The flexibility of the proteins is reflected by atomic displacement parameters (B-factors), which are considerably larger than for small molecules at 298 K. Thus, an optimal deconvolution of deformation density and thermal motion is not guaranteed, which prevents a free refinement of MP parameters but allows an application of transferable, fixed MP parameters. The analysis of the topological properties, such as the density at bond critical points (BCPs) and the Laplacian, reveals systematic differences between static and dynamic EDs. Zero-point-vibrations, yet present in dynamic EDs at low temperature, affect but marginally the EDs of small molecules. The zero-point-vibrations cause a smearing of the ED, which becomes more pronounced with increasing temperature. Topological properties, primarily the Laplacian, of covalent bonds appear to be more sensitive to effects by temperature and the polarity of the bonds. However, dynamic EDs at ca. 20 K based on MP models provide a good characterization of chemical bonding. Both the density at BCPs and the Laplacian of hydrogen bonds constitute similar values from static and dynamic EDs for all studied temperatures. Deformation densities demonstrate the necessity of the employment of MP parameters in order to comprise the nature of covalent bonds. The character of hydrogen bonds can be roughly pictured by IAM, whereas MP parameters are recommended for a classification of hydrogen bonds beyond a solely interpretation of topological properties.

## Introduction

A model based on independent spherical atoms (IAM) suffices to describe a crystal structure. However, this model does not take into account the redistribution of valence electrons due to effects of chemical bonding. The integrated intensities of Bragg reflections are usually well described by the IAM, but a discrepancy remains between model and data, especially for accurate diffraction data measured at low temperatures up to high resolution. This gap allows the true electron density distribution to be determined by X-ray diffraction. In turn, analysis of such densities will reveal properties of chemical interactions.

Electron densities beyond the IAM can be described by the multipole model according to *Hansen* and *Coppens*.^1^ The multipole model is an extension of the IAM, introducing a multitude of parameters (the multipole parameters) for describing the relatively small differences between true and IAM electron densities. While a multipole refinement is the established approach for describing electron densities of small molecules,^2^–^4^ only a few proteins have been subject to electron-density studies based on multipole models.^5^–^9^ As an alternative, transferable multipole parameters from a data base^10^–^17^ can be employed without refinement. In this approach, a suitable pseudo-atom with its associated multipole parameters – e.g. as determined for small molecules – is assigned to every atom in the structure. Thus, aspherical atomic form factors replace the spherical form factors of independent atoms, but the refineable parameters are the same as for the IAM. The multipole model implies a deconvolution of the effects of thermal motion and chemical bonding on the electron density. The usual interpretation of structure models is based on these deconvoluted, static densities. However, atomic vibrations are present in all crystals, even at zero temperature, and their effects on electron densities and properties of crystallized compounds cannot be switched off. Atomic displacements are particularly prominent for proteins, because they are often related to their function and chemical properties, which can require a particular degree of flexibility.^18^ Crystallographic B-factors of proteins reflect, apart from thermal vibrations and other factors, the degree of its flexibility. It is proposed that the understanding of chemical properties of proteins, with regard to functional inferences, may be enhanced by consideration of dynamic densities in addition to static densities.^19^,^20^

In the present contribution we summarize the method of computation of dynamic electron densities of multipole models and IAMs. An overview is given of the features of dynamic electron densities at very low temperatures in comparison to static electron densities for several amino acids and a tripeptide, especially concentrating on topological properties.^19^,^21^ The previously published multi-temperature study of dynamic electron densities of d,l-serine is reviewed.^19^,^22^ These results are then used for a discussion of chemical bonding in the protein crambin [Protein Data Bank^23^ (PDB) reference: 3nir] on the basis of static and dynamic densities derived from X-ray diffraction data at 100 K published by *Schmidt* et al.^6^ In accordance with a parallel study on hen egg-white lysozyme HEWL,^20^ we find that atomic displacement parameters (ADPs) assume values in crambin at 100 K that are comparable to or larger than values of ADPs of small molecules at room temperature. It is shown that the dynamic densities of crambin exhibits features comparable to those of serine at 298 K. Consequences for the application of multipole refinements to proteins are discussed.

## Methods and Calculations

### Dynamic Model Densities

The IAM describes a crystal structure by its refined atomic positions and atomic displacement parameters (ADPs), and considers a spherical atomic density distribution. In addition to the IAM, a high-order refinement of the IAM (IAM-HO) leads to an improved deconvolution of static density and atomic displacements.^24^ ADPs describe, among other effects, the displacement of the atoms about their positions due to thermal motion and/or static disorder. Atomic displacement parameters can either be presented as tensor *U*_ij_ or as isotropic B-factors B_eq_ = 8π^2^*U*_eq_. In addition to the coordinates and ADPs of a crystal structure, a multipole model employs multipolar extensions of the atomic density. These expansions comprise a spherical entity which describes the core density and part of the valence density and an aspherical valence density considering the redistribution of the density due to chemical bonding. Such a multipole model can be refined freely with respect to its population coefficients, radial functions and expansion/contraction parameters of the radial functions, if appropriate X-ray diffraction data are available. Alternatively, transferable multipole parameters from various databases^10^–^17^ are available that improve the atomic scattering factors. As opposed to atomic form factors of spherical atoms, these database parameters consider the chemical environment of each atom and they may be kept fixed during structure refinement. The multipolar extensions lead to a deviation of the aspherical-atom density from the IAM density.

Basing on IAM and multipole models, dynamic electron densities [*ρ*^IAM^_dyn_(**r**) and *ρ*^MP^_dyn_(**r**), respectively] can be constructed by inverse Fourier transform of the structure factors of the respective models (Fourier map), employing the method of Fast Fourier Transform (FFT).^19^ This procedure has been implemented in the computer program PRIOR.^19^,^25^ It has been demonstrated that accurate *ρ*^IAM^_dyn_(**r**) and *ρ*^MP^_dyn_(**r**) are obtained, if volumetric pixels associated with *ρ*(**r**) are chosen of a sufficiently small size (0.04 Å), which corresponds to reflections up to at least [sin(*θ*)/*λ*]_max_ = 6 Å^–1^. Although far beyond the accessible experimental limit and despite many very weak reflections, the large number of reflections is necessary for obtaining dynamic electron densities free of series-termination effects. Static electron densities, *ρ*^IAM^_stat_(**r**) and *ρ*^MP^_stat_(**r**), are produced by employment of atomic form factors of spherical and aspherical atoms, respectively, and thus do not include atomic displacement parameters. While static electron densities are deconvoluted from atomic displacements, dynamic electron densities display a time-averaged crystal structure.

Deformation densities can be generated to visualize the difference between the density based on the multipole model and the promolecule IAM density. For the compounds described in the present contribution, dynamic deformation densities are obtained by subtraction of the dynamic IAM density from the corresponding multipole model density:^19^


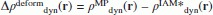
(1)

where IAM* is the IAM obtained by setting to zero all multipole parameters in the multipole model.

A topological analysis of the static and dynamic electron densities based on IAM and multipole models allows both a qualitative and quantitative interpretation. Critical points, such as local maxima or saddle points of the density, can be obtained. Local maxima of the density indicate atomic maxima. Saddle points correspond to bond critical points (BCPs), and their presence indicates chemical interactions. The density at BCPs, *ρ*(BCP), and its second derivative, ^2^*ρ*(BCP) (Laplacian), provide information on chemical bonding. A negative Laplacian, along with a large value of *ρ*(BCP), indicates a concentration of density at the BCP which would be expected for shared-shell interactions such as covalent bonds. A depletion of the density at the BCP is suggested by a positive Laplacian together with a small value of *ρ*(BCP). Charge depletion is typically observed for closed-shell interactions such as hydrogen bonds, van der Waals contacts and ionic interactions. Charge-shift bonds are represented by either a slightly negative or a positive Laplacian and a large value of *ρ*(BCP).^26^ Topological properties of static electron densities described in the present work were obtained by VMoPro.^28^ Dynamic electron densities were topologically analyzed by EDMA.^29^

### Computational Details of Crambin

The structure model of crambin published by *Schmidt* et al.^6^ (PDB: 3nir) was employed as starting model for the structure refinement. The crystallographic data of crambin (PDB: 3nir)^6^ are summarized in Table 1. After solvent correction of the structure factors by the flat bulk solvent method^30^,^31^ a refinement of the independent spherical atom model (IAM) against X-ray diffraction data was performed with the computer program MoPro.^28^ Initially, the scaling factor, the coordinates and the atomic displacement parameters (ADPs) of ordered, non-hydrogen atoms with a temperature factor B < 8 Å^2^ were refined alternately against all reflections. Hydrogen atoms were fixed at bond lengths known from neutron diffraction experiments^32^ and their ADPs constrained to values 1.2 or 1.5 times the values of their parent atoms. After convergence, a IAM refinement against high-order (IAM-HO) reflection data was performed in order to achieve an optimal deconvolution of the static electron density from the atomic displacements.^6^,^8^,^24^ For that purpose, the structure model was refined against reflections in the resolution range of 0.5 to 1.0 Å to convergence. Subsequently, solely the scaling factor was refined against all data, and a complete set of structure factors and the final IAM-HO were obtained. Agreement indices of the refinement steps are given in Table 1. Using the final IAM-HO, *ρ*^IAM^_stat_(**r**) was generated by superposition of atomic densities by VMoPro.^28^ Employing the coordinates and ADPs from the final IAM-HO, the computer program PRIOR^19^ was used to reconstruct *ρ*^IAM^_dyn_(**r**) on a grid of 576 × 512 × 1024 pixels. The number of pixels corresponds to a grid size of 0.04 Å, which ensures the absence of series termination effects.^19^

**Table 1:** Crystallographic data of crambin (PDB: 3nir)^6^ and agreement indices (0.48–20 Å) of the refinements of the present work. Values of the residual densities Δ*ρ*_min/max_ from (*F*_o_–*F*_c_)-maps of the three structure models are given for the plane of the salt bridge (OXT_46–Nε_10–Nη2_10)

Space group	*P*2_1_
Z	2
*a* /Å	22.33
*b* /Å	18.47
*c* /Å	40.77
*β* /°	90.55
*V* /Å^3^	16813.95
*T* /K	100
[sin(*θ*)/*λ*]_max_ /Å^–1^	1.04
*d_mi_*_n_ /Å	0.48
Completeness /%	97
Redundancy	3.7
Unique reflections	156860

**Agreement indices:**	
**IAM**	
*R*_F_ /%	13.77
*wR*_F_ /%	15.84
Δ*ρ*_min/max_ /electrons**·**Å^–3^	–0.424/0.275
**IAM-HO**	
*R*_F_ /%	14.46
*wR*_F_ /%	16.59
Δ*ρ*_min/max_ /electrons**·**Å^–3^	–0.560/0.302
**ELMAM2-model**	
*R*_F_ /%	13.83
*wR*_F_ /%	15.79
Δ*ρ*_min/max_ /electrons**·**Å^–3^	–0.552/0.275

For a multipole model, multipole parameters from the ELMAM2-database^17^ were transferred to the final IAM-HO (ELMAM2-model). The scaling factor, the coordinates and the ADPs of ordered, non-hydrogen atoms with a temperature factor B < 8 Å^2^ were refined alternatingly against all reflections (MoPro^28^). During that refinement the multipole parameters were fixed. It is accepted that a free refinement of multipole parameters is only reasonable with small ADPs present.^33^ In the Section “Electron Densities of Crambin” of the present contribution it is demonstrated that the ADPs of crambin are considerably larger in comparison to small molecules. Thus, a free refinement of multipole parameters did not appear to be reasonable and has not been performed for crambin. This may explain the difference between the *R*-values obtained in the present refinement (Table 1) and the *R*-value published by *Schmidt* et al.,^6^ where after a free refinement of multipole parameters an *R*-value of 12.7 % was obtained. *ρ*^MP^_stat_(**r**) was generated by superposition of aspherical atomic densities by VMoPro.^28^ *ρ*^MP^_dyn_(**r**) was constructed by the computer program PRIOR,^19^ (576 × 512 × 1024 volumetric pixels) using the coordinates, the ADPs and the multipole parameters. To generate the dynamic deformation density, Δ*ρ*^deform^_dyn_(**r**) [Equation (1)], the atomic coordinates and ADPs of the multipole model of crambin were used to compute *ρ*^IAM^*_dyn_(**r**). Topological properties were obtained by EDMA.^29^ The one-to-one correspondence between critical points in the static and dynamic densities of crambin provide evidence for the absence of series-termination effects in the latter.

## Discussion

### Electron Densities of Small Molecules

Static and dynamic electron densities at ca. 20 K of α-glycine,^34^ l-alanine,^35^ d,l-serine^22^ and the tripeptide alanyl-tyrosyl-alanine (Ala-Tyr-Ala)^36^ have been reconstructed and analyzed in the literature.^19^,^21^ These studies comprised the analysis of electron densities based on IAM, IAM-HO, Invariom model (transferable multipole parameters from the Invariom-database^13^), and the multipole (MP) model. We summarize the major findings of references^19^,^21^ herein.

The topological analyses of the small molecule densities^19^,^21^ show good agreement between positions of local maxima of non-hydrogen atoms found in dynamic electron densities and corresponding static densities. For dynamic densities, hydrogen atoms may be included in atomic basins of their parent atoms due to atomic displacements,^37^ which is true for most of the hydrogen atoms of the small molecules discussed in the present contribution.^19^,^21^ The comparison of density values at local maxima from dynamic and static densities indicates that the dynamic density is smeared near the nuclei, which is apparent as lower density values. In dynamic densities at ca. 20 K, atomic displacements remain as zero-point vibrations, which can be held responsible for the lower density values at local maxima.

Dynamic deformation densities Δ*ρ*^deform^_dyn_(**r**) [Equation (1)] feature a similar appearance of covalent bonds as in corresponding static deformation densities. This qualitative agreement is complemented by inspection of the positions of BCPs and their associated topological properties. In dynamic densities all covalent bonds and hydrogen bonds possess BCPs, which are located at positions matching the corresponding positions of BCPs in static densities. The comparison of *ρ*(BCP) of chemical bonds from static and dynamic densities at ca. 20 K indicated that zero-point-vibrations lead also to a smearing of the density at BCPs. For dynamic densities, *ρ*(BCP) of covalent bonds is generally slightly, but systematically, smaller than from static densities. With increasing polarity of covalent bonds, differences between dynamic and static densities become more manifest. As opposed to covalent bonds, hydrogen bonds from dynamic densities constitute systematically slightly larger values of *ρ*(BCP) than from static densities. This observation may be explained with the fact, that smearing of a density from high-density regions leads to increased values in low-density regions. The dynamic deformation densities exhibit similar features of hydrogen bonds compared with static deformation densities. These results suggest that low-temperature dynamic densities can be employed as a good approximation to static densities, when analyzed according to the quantum theory of atoms in molecules (QTAIM)^38^ in order to describe chemical bonding.

The study of low-temperature densities was complemented by electron densities of d,l-serine at 20, 100, and 298 K.^19^,^22^ Density values near local maxima from dynamic densities at higher temperatures are even more lower than the values from dynamic densities at ca. 20 K. Thus, increased thermal motion at higher temperatures, which is far beyond zero-point vibrations, causes a more pronounced smearing of the density close to local maxima. Compared to BCPs from static densities, BCPs of all covalent bonds and all hydrogen bonds were obtained from dynamic densities. *ρ*(BCP) is hardly affected by temperature and the values from dynamic densities show good agreement with corresponding values from static densities. However, differences of ^2^*ρ*(BCP) of covalent bonds, comparing static and dynamic densities, become more apparent with both increasing temperature and with increasing polarity of the bond studied. ^2^*ρ*(BCP) of non-polar or slightly polar bonds show good agreement between dynamic and static densities at 20 and 100 K. However, for C–O and C–N bonds from densities at 298 K strong variations of ^2^*ρ*(BCP) are observed. The topological characterization of polar bonds such as C–O bonds appeared not to be straightforward, which has also been reported earlier for static electron densities.^39^–^42^ In low-density regions, as present for hydrogen bonds, static and dynamic densities show similar values for properties *ρ*(BCP) and ^2^*ρ*(BCP) within the complete temperature range studied.

The comparison of densities based on IAM and multipole models showed larger values for *ρ*(BCP) of covalent bonds for the latter, while the opposite is true for hydrogen bonds. It confirms that it is imperative to introduce multipole parameters to reveal the character of covalent bonds. A simple characterization of hydrogen bonds does not categorically require the employment of multipole parameters, while it is strongly recommended for the purpose of correlating bond lengths of hydrogen bonds with their topological properties at BCPs.^42^,^43^ Topological properties of hydrogen bonds can be accurately characterized by *ρ*_INV_.

### Electron Densities of Crambin

**Figure 1:**
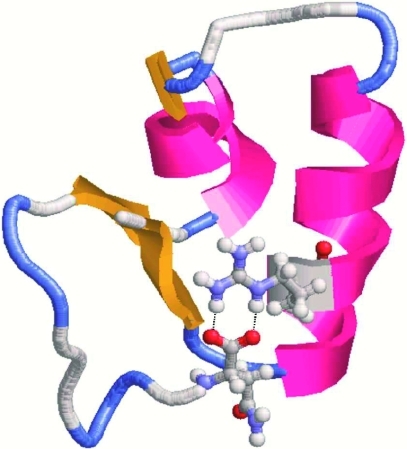
Schematic drawing of Arg10 and Asn46 in crambin. Dotted lines indicate the salt bridge.

The small protein crambin (PDB: 3nir)^6^ offers one of the most extensive X-ray diffraction data of highest resolution available for proteins, and is thus the most suitable protein for electron-density studies. The biological function of crambin function is an open issue. In crambin, an ion pairing through hydrogen bonding between the guanidinium group of the arginine residue Arg10 and the carboxyl group of the C-terminal asparagine residue Asn46 has been described^44^ (Figure 1). This salt bridge is reported as promoting the formation of the stabilizing disulfide bonds in crambin and contributing to its stability and well-ordered structure.^45^ In the present work, the focus rests on the two amino acids, involved in the formation of the salt bridge, to investigate the correlations between static densities, dynamic densities, and chemical bonding. Dynamic densities based on IAM and ELMAM2^17^ models of crambin (PDB: 3nir)^6^ have successfully been computed and are compared with corresponding static densities.

**Figure 2:**
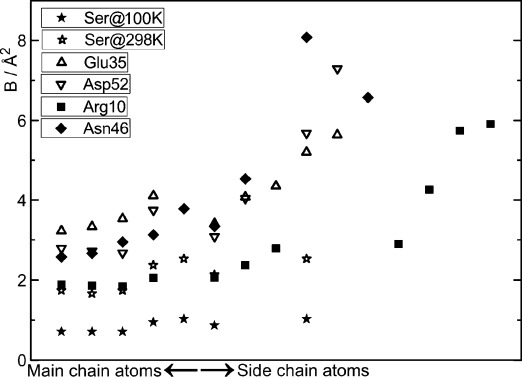
B-factors of Arg10 (squares), Asn46 (diamonds) from crambin, corresponding B-factors of Glu35 (up triangles) and Asp52 (down triangles) from HEWL^20^ and corresponding B-factors from d,l-serine at 100 K (filled asterisks) and at 298 K (open asterisks).^19^

A deconvolution of thermal motion and deformation density is presumed by the multipole model. Hence, the magnitude of atomic displacement factors is of tremendous importance for a meaningful interpretation of an electron density. On this account, B-factors of crambin are inspected and compared with corresponding values of a d,l-serine^19^,^22^ at different temperatures. Asn46, which is located at the C-terminal loop of crambin, shows substantially larger B-factors at 100 K than d,l-serine at 298 K^19^ (Figure 2). A large difference between the B-factors of Arg10 at 100 K and d,l-serine at 100 K^19^ can be observed (Figure 2). The B-factors of Arg10 have more or less the same magnitude as corresponding values of d,l-serine at room temperature,^19^ which would be considered as being too large for a free refinement of multipole parameters or a typical electron density study. Substantially larger B-factors in comparison with d,l-serine^19^ have also been observed for HEWL (Figure 2).^20^ The B-factors of main-chain atoms in crambin are for the most part smaller than the corresponding values of HEWL.^20^ Thus, it may be inferred that crambin is less flexible than HEWL^20^ and that the effect of atomic displacements on the density is less pronounced for crambin. For proteins at 100 K the entity of atomic displacement parameters may be regarded less as thermal motion but mainly as frozen disorder, which reflect the intrinsic flexibility of the protein.^20^ The large B-factors of the two proteins, compared with the amino acid, underline the potential existence of basic differences between proteins and small molecules due to an intrinsic flexibility of proteins as specified for HEWL.^20^

Irrespective of their origins, large ADPs hamper an optimal deconvolution of thermal motion and deformation density and thus a meaningful refinement of multipole parameters. What remains is the possibility to employ transferable multipole parameters from a database^10^–^17^ without refinement. This approach has been shown to lead to an improved deconvolution of electron density and thermal motion for small molecules at room temperature.^22^,^46^ According to the *R*-values and the values for the residual densities Δ*ρ*_min/max_ of the refinement of crambin (Table 1), the ELMAM2-model does not provide a better fit to the diffraction data than the IAM does, which will originate in the large ADPs. However, ADPs from the ELMAM2-model of crambin are usually smaller than those from the IAM or IAM-HO. The improved structure parameters indicate that, despite slightly larger *R*-values and residual densities (Table 1), the crystal structure based on multipole parameters is closer to the true structure of crambin than the IAM or IAM-HO. Thus, the employment of fixed multipole parameters from a database provides a more accurate protein structure and is preferable compared to the IAM.

**Figure 3:**
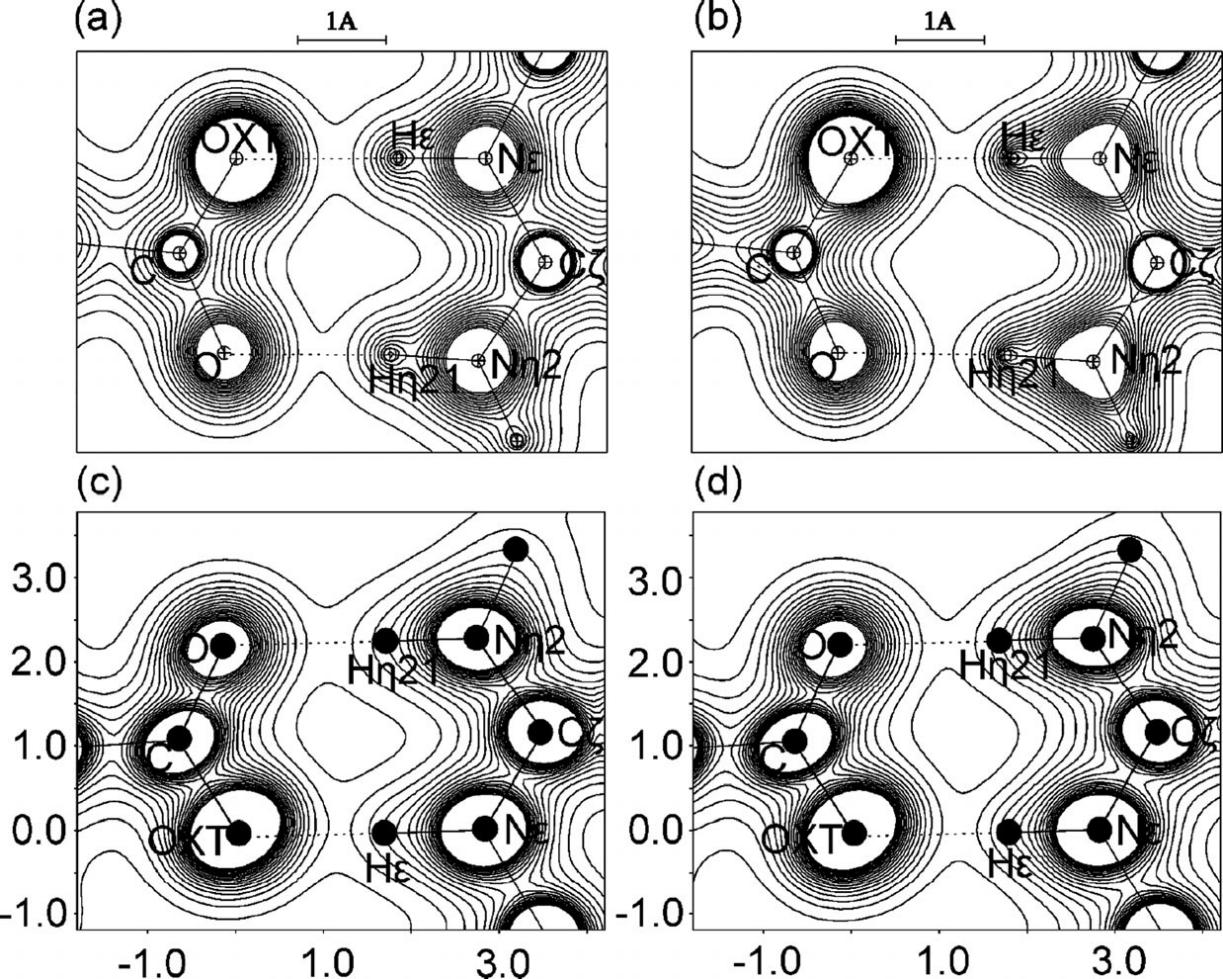
Sections of 6 × 5 Å^2^ through the plane of atoms (OXT_46–Nε_10–Nη2_10) involved in the salt bridge. (a) Static density based on the IAM, (b) static density based on the multipole model, (c) dynamic density based on the IAM, and (d) dynamic density based on the multipole model. Numbers on the axes refer to the scale in Å. Contour lines of equal density are given from 0.2–3.5 electrons/Å^3^ in steps of 0.2 electrons**·**Å^–3^.

The dynamic densities in the region of the salt bridge in crambin exhibit an elliptical distortion, as opposed to the static densities, which feature more or less spherically shaped atomic contour lines (Figure 3). The distortion arises from anisotropic atomic displacements present in dynamic densities but not considered in static densities.^20^ Discrete maxima of hydrogen atoms can be determined for static densities, while in dynamic densities hydrogen atoms are encompassed by the atomic basins of the respective parent atoms. This feature is typically observed for dynamic densities of small molecules and proteins.^[19, 20, 21, 37, 42]^

**Figure 4:**
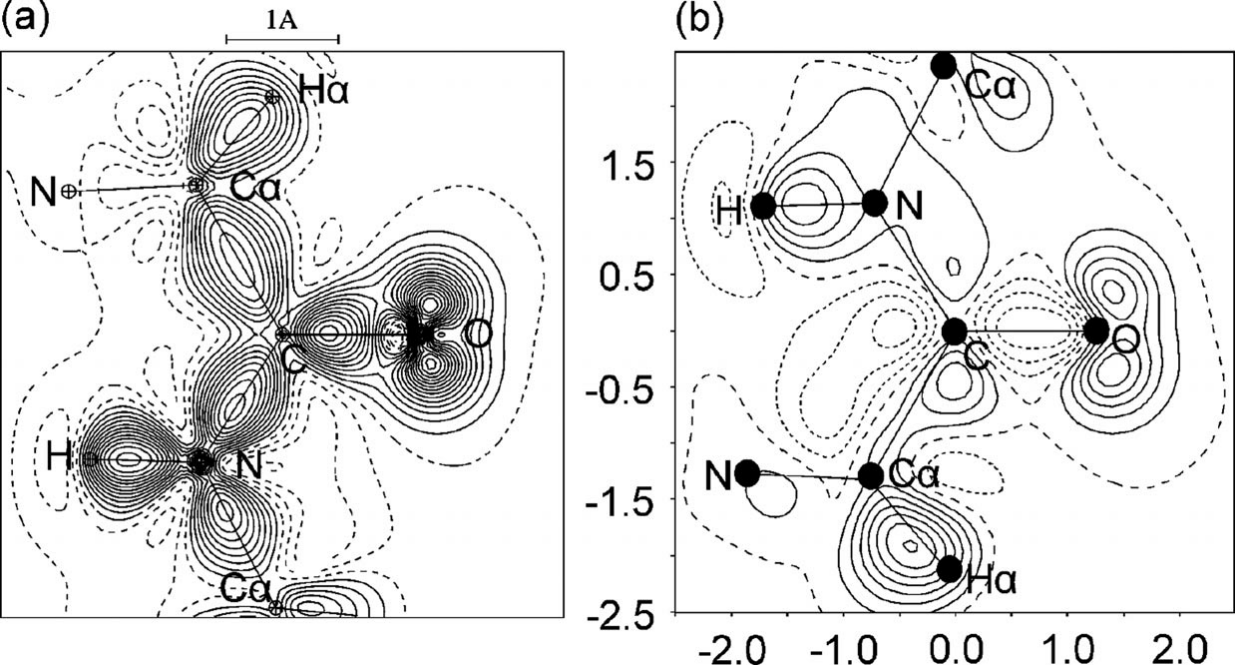
Sections of 5 × 5 Å^2^ through the peptide bond plane C_9–O_9–N_10 from Ala9 to Arg10. (a) Static deformation density, (b) dynamic deformation density Δ*ρ*^deform^_dyn_(**r**). Numbers on the axes refer to the scale in Å.Contour lines of equal density are given in intervals of 0.05 electrons**·**Å^–3^.

The qualitative comparison of the static densities *ρ*^IAM^_stat_(**r**) and *ρ*^MP^_stat_(**r**) of crambin (Figure 3) indicates small differences due to employment of multipole parameters. The consideration of chemical bonding causes a deviation of the density contours compared to the IAM density, especially apparent for the nitrogen atoms of the displayed plane (Figure 3). Furthermore, a closer inspection of the static densities indicates a larger amount of density in the region of the BCPs of the covalent C–N and C–O bonds for *ρ*^MP^_stat_(**r**). The static deformation density [Figure 4(a)] reveals a clear effect of chemical bonding on the density, which allows lone-pairs of the oxygen atom and an accumulation of density in the region of covalent bonds to be observed. In contrast, the dynamic deformation density Δ*ρ*^deform^_dyn_(**r**) [Figure 4(b)] does not exhibit structure as pronounced as the static deformation density does. The deformation density representing the lone-pairs of the oxygen atoms is strongly attenuated and there is no pronounced accumulation of electron density for covalent bonds visible. As reported for HEWL at 100 K,^20^ the effects by chemical bonding are overlapped by atomic displacements and thus they cannot be revealed in the dynamic densities of crambin at 100 K. Since intensities of Bragg reflections directly correspond to dynamic electron densities, this similar appearance of dynamic densities of IAMs and multipole models questions the possibility of deconvoluting the effects of thermal motion and chemical bonding. The evaluation of the B-factors of the atoms in crambin supports the interpretation that predominating atomic displacements impede the observation of effects by chemical bonding as reported for HEWL.^20^

### Topological Properties of Electron Densities of Crambin

#### Covalent Bonds

Topological properties of covalent bonds in crambin are given in Table 2 and Table 3 and complement quantitatively the electron densities plots in Figure 3 and Figure 4. The dynamic densities are subject to smearing, which is observable as distortions of the dynamic densities (Figure 3). For this reason, *ρ*^MP^_stat_(BCP) of covalent bonds are larger than *ρ*^MP^_dyn_(BCP). This feature is noticed also for small molecules at different temperatures^19^,^21^,^42^ and for HEWL.^20^ The comparison of the densities based on IAM shows that *ρ*^IAM^_stat_(BCP) is either of similar value as or smaller than *ρ*^IAM^_dyn_(BCP).

**Table 2:** Topological properties of covalent bonds in Arg10 from static and dynamic densities. First line: *ρ*(BCP) /electrons·Å^–3^, second line: ∇^2^*ρ*(BCP) /electrons·Å^–5^

Bond	IAM-HO static	dynamic	MP static	dynamic
C–O	2.121	2.407	2.684	2.115
	5.71	35.60	–25.45	39.94
Cγ–Cδ	1.194	1.226	1.635	1.275
	1.33	5.56	–9.64	6.15
Cα–C	1.185	1.164	1.615	1.276
	1.34	0.64	–9.16	–1.29
Cα–Cβ	1.183	1.187	1.598	1.108
	1.44	1.55	–8.97	3.28
Cβ–Cγ	1.183	1.178	1.561	1.193
	1.44	2.01	–8.47	1.87
C–N_11	1.802	1.872	2.301	1.920
	–3.89	15.41	–23.75	11.37
Cζ–Nη1	1.863	2.253	2.454	1.965
	–5.11	29.01	–27.49	22.64
Cζ–Nη2	1.754	1.752	2.398	1.743
	–2.77	18.50	–24.51	15.41
Cζ–Nε	1.775	1.838	2.439	1.679
	–3.21	19.25	–26.52	21.37
C_9–N	1.766	1.931	2.241	1.930
	–3.18	18.76	–21.59	14.51
Cα–N	1.464	1.482	1.733	1.490
	2.18	7.24	–9.48	6.38
Cδ–Nε	1.461	1.488	1.715	1.446
	2.23	10.56	–9.66	13.06
Cα–Hα	1.204	–	1.870	–
	–3.82	–	–18.65	–
Cβ–Hβ2	1.221	–	1.802	–
	–4.10	–	–16.95	–
Cβ–Hβ3	1.221	–	1.803	–
	–4.10	–	–16.95	–
Cγ–Hγ2	1.221	–	1.803	–
	–4.10	–	–16.94	–
Cγ–Hγ3	1.221	–	1.803	–
	–4.10	–	–16.94	–
Cδ–Hδ2	1.219	–	1.866	–
	–4.10	–	–18.64	–
Cδ–Hδ3	1.219	–	1.867	–
	–4.09	–	–18.63	–
N–H	1.567	–	2.297	–
	–8.68	–	–39.10	–
Nε–Hε	1.567	–	2.326	–
	–8.64	–	–40.55	–
Nη1–Hη11	1.567	–	2.227	–
	–8.70	–	–36.47	–
Nη1–Hη12	1.568	–	2.289	–
	–8.67	–	–39.66	–
Nη2–Hη21	1.566	–	2.289	–
	–8.69	–	–39.69	–
Nη2–Hη22	1.566	–	2.290	–
	–8.68	–	–39.71	–

**Table 3:** Topological properties of covalent bonds in Asn46 from static and dynamic densities. First line: *ρ*(BCP) /electrons·Å^–3^, second line: ∇^2^*ρ*(BCP) /electrons·Å^–5^

Bond	IAM-HO static	dynamic	MP static	dynamic
Cγ–Oδ1	2.166	2.949	2.799	2.713
	9.08	20.91	–18.33	30.80
C–O	2.073	3.260	2.714	3.018
	2.68	34.35	–32.53	38.19
C–OXT	2.044	2.221	2.723	1.962
	8.91	30.43	–32.07	36.74
Cβ–Cγ	1.230	1.409	1.637	1.398
	9.40	14.78	–9.70	15.56
Cα–Cβ	1.185	1.162	1.469	1.167
	1.43	3.25	–7.60	1.28
Cα–C	1.159	1.379	1.675	1.319
	1.54	14.73	–10.97	15.58
Cγ–Nδ2	1.819	1.884	2.309	2.010
	–4.19	22.04	–23.61	22.27
C_45–N	1.795	1.685	2.285	1.720
	–3.79	11.51	–23.14	9.84
Cα–N	1.467	1.520	1.939	1.592
	2.12	11.30	–11.17	8.37
Cα–Hα	1.205	–	1.873	–
	–3.82	–	–18.63	–
Cβ–Hβ2	1.221	–	1.801	–
	–4.10	–	–16.96	–
Cβ–Hβ3	1.221	–	1.801	–
	–4.10	–	–16.96	–
Nδ2–Hδ21	1.567	–	2.284	–
	–8.68	–	–39.49	–
Nδ2–Hδ22	1.565	–	2.284	–
	–8.71	–	–39.47	–
N–H	1.567	–	2.317	–
	–8.68	–	–40.12	–

For covalent bonds, *ρ*^IAM^_stat_(BCP) are systematically lower than *ρ*^MP^_stat_(BCP), a feature which has also been observed for small molecules^19^,^21^ and HEWL^20^ (Table 2 and Figure 3). This supports the inference, drawn from the static deformation density of crambin [Figure 4(a)], that chemical bonding in proteins is only revealed by employment of the multipole model. In contrast to static densities, *ρ*^IAM^_dyn_(BCP) and *ρ*^MP^_dyn_(BCP) of crambin do not show systematic differences at covalent bonds, except for C–O bonds. Values of *ρ*^IAM^_dyn_(BCP) of C–O bonds are larger than *ρ*^MP^_dyn_(BCP). This observation is also true for C–O bonds in HEWL^20^ and may reflect that polar bonds often show a peculiar behavior.^[19, 21, 39–42]^ Similar values of *ρ*^IAM^_dyn_(BCP) and *ρ*^MP^_dyn_(BCP) is visualized by the dynamic deformation density Δ*ρ*^deform^_dyn_(**r**), which exhibits attenuated features as compared to static deformation densities (Figure 4). It has been proposed for HEWL at 100 K that effects by chemical bonding are masked in dynamic densities by large atomic displacements.^20^ Nevertheless, the presented results for crambin and HEWL^20^ suggest that dynamic electron densities based on multipole models provide reasonable values of *ρ*(BCP) and thus allow a characterization of covalent bonds, like it has been found for small molecules.^19^,^21^

As opposed to *ρ*(BCP), the values of ^2^*ρ*(BCP) of covalent bonds show considerable differences between static and dynamic densities. While the Laplacian is strongly negative for covalent bonds from *ρ*^MP^_stat_(**r**) indicating a covalent character, ^2^*ρ*(BCP) is positive for all covalent bonds from dynamic electron densities of crambin. Covalent bonds from *ρ*^IAM^_stat_(**r**) possess either a positive or a slightly negative ^2^*ρ*(BCP). These results are on a par with the findings for HEWL at 100 K^20^ and for d,l-serine at 298 K,^19^ for which large ADPs have been correlated with positive values of ^2^*ρ*(BCP). The multi-temperature study of d,l-serine^19^ pointed out that topological properties, notably the Laplacian, are subject to strong variations with increasing temperature and polarity of covalent bonds. The meaning of topological properties of dynamic densities is still subject of research.

#### Hydrogen Bonds

**Figure 5:**
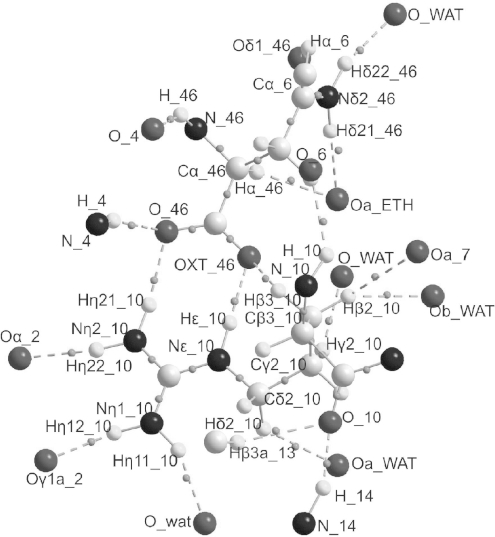
Schematic representation of hydrogen bonds formed (dashed lines) by the two amino acids Arg10 and Asn46 involved in the salt bridge. The small grey spheres indicate bond critical points.

The two amino acids forming the salt bridge via a pair of hydrogen bonds are involved in other hydrogen bonds too (Figure 5). Topological properties of hydrogen bonds, including the ones constituting the salt bridge, are summarized in Table 4 and Table 5. Although, values of *ρ*(BCP) of hydrogen bonds show similar values for static and dynamic densities, small but systematic differences can be observed and summarized as follows: *ρ*^IAM^_dyn_(BCP) > *ρ*^MP^_dyn_(BCP) > *ρ*^IAM^_stat_(BCP) > *ρ*^MP^_stat_ (BCP). This behavior has also been reported for hydrogen bonds in d,l-serine at all temperatures^19^ and in HEWL.^20^ It can be inferred in this regard that features of hydrogen bonds become pronounced by taking into account atomic displacements. A description of hydrogen bonds by the IAM may be regarded as inappropriate, since the IAM does not consider chemical bonding. However, as concluded from the study of *Mondal* et al.,^19^ a rough description of hydrogen bonds can be obtained from the IAM in case a correlation with the bond lengths is not considered.^47^,^48^ The Laplacians from static and dynamic densities possess positive values for the hydrogen bonds studied in crambin. Thus, the hydrogen bonds can be described by charge depletion at BCPs, which is typical for closed-shell interactions. In contrast to ^2^*ρ*(BCP) of covalent bonds in crambin, the Laplacian of hydrogen bonds does not exhibit large variations with temperature. As also reported for d,l-serine,^19^ hydrogen bonds in crambin can be topologically described by both static and dynamic electron densities.

## Conclusions

Large atomic displacement parameters, as present in dynamic densities of proteins at 100 K or small molecules at room temperature, prevent both a free refinement of multipole parameters and a better fit of the multipole model to the diffraction data than the fit of the IAM. However, employment of transferable multipole parameters from a database,^10^–^17^ which are fixed during the refinement, provides an improved description of the atomic scattering factors, and leads to more accurate electron densities than those of the IAM. Dynamic electron densities of small molecules and proteins based on IAM and multipole models can be successfully constructed with the computer program PRIOR^19^ without existence of series termination effects. These dynamic densities can be used as reference densities for an application of the Maximum Entropy Method (MEM) allowing the construction of dynamic densities, which are independent of a structural model and do not suffer from potential shortcomings imposed by it.^42^

**Table 4:** Topological properties of the salt bridge (denoted by *) and hydrogen bonds formed by Arg10 from static and dynamic densities. First line: *ρ*(BCP) /electrons·Å^–3^, second line: ∇^2^*ρ*(BCP) /electrons·Å^–5^

Bond	IAM-HO static	dynamic	MP static	dynamic
OXT_46**···**Hε_10–Nε_10*	0.282	0.356	0.258	0.295
	2.81	2.17	1.79	2.10
O_46**···**Hη21_10–Nη2_10*	0.230	0.294	0.182	0.243
	2.40	1.57	1.65	1.82
Oγ1a_2**···**Hη12_10–Nη1_10	0.263	0.309	0.204	0.260
	2.61	1.43	2.00	1.72
Oα_2**···**Hη22_10–Nη2_10	0.226	0.265	0.176	0.211
	2.33	1.51	1.57	1.63
O_10**···**H_14–N_14	0.216	0.244	0.175	0.192
	2.30	2.25	1.62	1.95
O_WAT**···**Hη11_10–Nη1_10	0.181	0.230	0.120	0.168
	2.02	1.65	1.59	1.82
O_6**···**H_10–N_10	0.100	0.123	0.078	0.094
	1.20	1.36	0.92	1.09
OXT_46**···**Hβ3_10–Cβ_10	0.077	0.093	0.055	0.066
	0.93	1.04	0.85	0.98
O_10**···**Hβ3a_13–Cβa_13	0.071	0.086	0.052	0.073
	0.86	0.98	0.81	1.01
Oa_7**···**Hβ2_10–Cβ_10	0.068	0.086	0.051	0.065
	0.83	0.96	0.75	0.91
O_WAT**···**Hγ2_10–Cγ_10	0.050	0.060	0.038	0.046
	0.63	0.71	0.49	0.57
Oa_WAT**···**Hδ2_10–Cδ2_10	0.037	0.053	0.023	0.036
	0.46	0.62	0.35	0.54
Ob_WAT**···**Hβ2_10–Cβ_10	0.031	0.043	0.021	0.031
	0.40	0.50	0.31	0.43

**Table 5:** Topological properties of hydrogen bonds formed by Asn46 from static and dynamic densities. First line: *ρ*(BCP) /electrons·Å^–3^, second line: ∇^2^*ρ*(BCP) /electrons·Å^–5^

Bond	IAM-HO static	dynamic	MP static	dynamic
Oa_ETH**···**HDδ21_46–Nδ2_46	0.245	0.320	0.172	0.251
	2.47	1.19	1.56	1.62
O_WAT**···**Hδ22_46–Nδ2_46	0.216	0.414	0.156	0.343
	2.30	–0.02	1.49	0.79
O_46**···**H_4–N_4	0.210	0.256	0.175	0.207
	2.24	2.24	1.54	1.96
O_4**···**H_46–N_46	0.174	0.206	0.140	0.160
	1.93	1.91	1.39	1.67
Oδ1_46**···**Hα_6–Cα_6	0.083	0.101	0.061	0.078
	1.01	1.12	0.96	1.15
Oa_ETH**···**Hα_46–Cα_46	–	–	0.037	0.057
	–	–	0.49	0.73

Atomic displacements are present in dynamic electron densities at low temperatures as zero-point vibrations, affecting the density but marginally.^19^ Proteins such as HEWL^20^ and crambin at 100 K have larger atomic displacement parameters (B-factors) than small molecules at room temperature. In agreement with results for HEWL,^20^ the B-factors of crambin at 100 K will mainly reflect frozen disorder and suggest an intrinsic flexibility which may be required for the function of a protein. The frozen disorder is visible as distortions of the electron density of crambin and HEWL.^20^ However, the atomic displacement parameters of crambin and HEWL are smaller than in most other protein crystals. Since static densities do not consider atomic displacements (flexibility), dynamic densities have to be considered in addition to static electron densities, in order to allow inferences regarding the function of a protein.

The insensitivity of *ρ*(BCP) to most of the effects comprised by atomic displacement parameters permits it to be a reliable topological descriptor of covalent bonds and hydrogen bonds from dynamic densities of small molecules and proteins in a large range of temperatures. In contrast to *ρ*(BCP), considerable variations of ^2^*ρ*(BCP) of covalent bonds can be observed with temperature, which become more apparent with increasing temperature and degree of polarity of the bonds. A theoretical description of the Laplacians of polar bonds, such as C–O bonds, in dynamic electron densities is not yet available. Nevertheless, topological entities from dynamic low-temperature densities of small molecules based on multipole models can accurately describe chemical bonds.^19^

Effects on electron densities by chemical bonding, revealed by multipole parameters, are visualized in the static deformation density. It is quantified by comparing topological properties of covalent bonds in static densities based on IAM and multipole models. As demonstrated for HEWL^20^ and crambin at 100 K, frozen disorder masks the effects of chemical bonding on dynamic electron density maps at 100 K. Thus, *ρ*(BCP) from dynamic densities based on IAM and multipole models exhibit similar values. On the other hand, both *ρ*(BCP) and ^2^*ρ*(BCP) of hydrogen bonds appear to be quite insensitive to the type of electron density map (static versus dynamic and IAM versus multipole model). Hydrogen bonds can therefore be well characterized by the topological properties of dynamic electron densities obtained from the multipole model with multipole parameters from a database.
